# Knowledge and Attitude of the Public Toward Bariatric Weight Loss Surgery and Its Impact on Candidates and Patients in the Al-Qassim Region, Saudi Arabia

**DOI:** 10.7759/cureus.50477

**Published:** 2023-12-13

**Authors:** Bilal S Al-Mushaigah, Rakan A Almesned, Osamah A Alsolai, Noor M Alfahhad, Abdulelah A Almesned

**Affiliations:** 1 College of Medicine, Qassim University, Buraydah, SAU; 2 College of Interventional Radiology, Qassim University, Buraydah, SAU; 3 College of Family Medicine, Qassim University, Buraydah, SAU; 4 Unaizah College of Medicine and Medical Sciences, Qassim University, Buraydah, SAU

**Keywords:** mental health, social impact, stigma, bariatric surgery, obesity

## Abstract

Background

Obesity is defined as abnormal or excessive fat accumulation that presents a serious health risk and is a major public health concern. Obesity prevention and management require evidence-based strategies that emphasize diet and physical activity. Bariatric surgery is also a life-changing procedure that can improve physical and mental health, but the stigma associated with it can prevent people from seeking treatment and affect their lives adversely. Studies have shown that bariatric surgery patients face discrimination from the public and healthcare professionals, which can lead to adverse psychological outcomes and hinder access to quality care.

Goals and methods

This study intends to explore the stigma related to bariatric surgery in Al-Qassim Region, Saudi Arabia, because it is crucial to understand its prevalence among the public and the influence it has on both those who have undergone the surgery and those who are considering it as an option. The participants had to complete an online questionnaire, comprised a general section and other sections based on whether or not the individual has, has not, or is considering bariatric surgery.

Results

A total of 988 individuals, 605 of whom were female (61.2%), agreed to participate in the study. The most common body mass index (BMI) category was 18.5-24.9 (43.5%, n=414). The majority of the participants had either agreed or strongly agreed that obesity is a disease (87.8%, n=867) and that genetic factors play a role in causing it (38.8%, n=383). The factors selected most commonly that increase the risk of obesity were “idle and lazy life” (76.5%, n=756) and “eating too much” (75.6%, n=747). Fewer than half of the participants (44.43%, n=439) reported that they had never thought about treating obesity through surgical operations, 9.62% (n=95) had considered it, and 3.74% (n=37) had actually undergone the surgery. Among those who underwent weight loss surgery (n=37), 43.20% (n=16) reported that they received critical comments or poor treatment from the community, 35.10% (n=13) felt ashamed or embarrassed to disclose their surgery, and 37.80% (n=14) avoided social situations or events because of those comments or poor treatment. The comments reported most often were “You have taken the easy way out instead of adopting a healthy lifestyle” (51.40%, n=19) and “Why didn’t you try to go on a diet?” (51.40%, n=19). Among those who have intentions to undergo weight loss surgery (n=95), a significant proportion of the participants (43%, n=40) agreed or strongly agreed that concerns about public opinion or community treatment could affect their decision to undergo weight loss surgery. Moreover, 32.6% (n=31) of them agreed or strongly agreed that society has a negative attitude toward individuals who have undergone obesity treatment. When asked whether they had ever avoided telling people that they were considering surgery because of potential adverse reactions, 42.10% (n=40) of the participants responded that they had.

Conclusion

This study helped bring attention to, and prove, the stigma related to bariatric surgery in Al-Qassim Region. Such stigma has prevented patients from seeking or undergoing a surgical option to manage their weight, even if it is the option recommended for them. As such, public education and awareness campaigns are encouraged to help reduce the stigma, as well as improve access to bariatric surgery for those who need it.

## Introduction

Being overweight and obesity are defined as abnormal or excessive fat accumulation that presents a serious health risk. A body mass index (BMI) of 25 or more is considered overweight, while 30 or more is obese [1]. Within the past several decades, there has been a significant increase in the prevalence of obesity [1,2]. Obesity is a major public health concern that is associated with numerous health problems. Further, it has been proven not to be attributable to a sole factor, but rather to complex interactions among multiple factors [3,4]. As it is a major concern that affects the world’s population, there is no doubt that techniques for every individual to manage it should be investigated.

Particularly, these techniques can be divided into two major categories: non-surgical (natural and medicinal) and surgical [2,5,6]. There are multiple methods in the non-surgical approach, natural (diet and/or exercise), or medicinal [2,5,6]. While these methods are less invasive, a major role in their success depends upon the willingness and determination of the individuals who opt for them [2].

As diets require either reducing caloric intake or portion sizes primarily and other diets focus on a particular nutrient (i.e., keto), they do not necessarily lead to sustained weight loss and require close follow-ups [2,5,6]. With respect to exercise, physical activity in general has multiple positive effects on the body and mental health of those who practice it. An eight-year weight loss study, the Look AHEAD study, was performed to assess the effects of intentional weight loss in adults with type 2 diabetes. Its results showed that, of those who engaged in intense lifestyle modifications, 34.5% had achieved a weight loss of 10% or more in the first year, and of these participants, 39.3% had maintained the 10% or more loss at year eight [7].

With respect to the medicinal approach, anti-obesity medications have been recommended in recent years for patients who are classified as obese or have had obesity-related complications. However, the weight loss is sustained only for as long as the medication is used [2,8].

Another method, the surgical approach, is also a viable option. Ongoing studies and published research have demonstrated that it is a safer procedure overall, with better effects, which has made surgeons more comfortable with recommending this method of treating obesity [2,5]. However, from a medical standpoint, although a number of studies have proven this surgery’s efficacy, patients who undergo this life-changing procedure may face the stigma surrounding it [5,9,10]. This causes those who may benefit more from this surgery, and perhaps need it, to avoid the surgery altogether, and causes those who have undergone it to feel bad after they face such situations post-surgery.

The goal of this study is to explore the stigma associated with bariatric surgery in the Al-Qassim Region because it is crucial to understand the prevalence of these stigmas among the public and the effect it has on both those who have undergone the surgery and those who are considering it as an option. Further, this can help break down barriers and increase access to life-changing treatments for those who need them.

## Materials and methods

The study employed a cross-sectional design, utilizing a self-administered questionnaire for data collection. The research was conducted in the Al-Qassim Region over a period of 12 months. To determine the sample size, Yamane’s Formula was applied, considering a target population of 1.52 million persons and a 5% margin of error, resulting in a required sample size of 399. Convenience sampling was chosen as the sampling technique, allowing for the quick and efficient collection of data from participants meeting the inclusion criteria: adults aged 18 or older residing in the Al-Qassim Region. Exclusion criteria comprised individuals under 18 years old and non-residents in the Al-Qassim Region.

The data collection involved the distribution of a self-administered questionnaire through online platforms, such as social media and forums. The questionnaire comprises multiple sections addressing demographics, perceptions of obesity, and attitudes toward bariatric surgery. Participants are queried about their beliefs regarding the causes of obesity, societal views on individuals who undergo bariatric surgery, and their personal experiences after the operation. The questionnaire further explores concerns about public opinion influencing the decision to undergo surgery and opinions on the effectiveness of bariatric surgery compared to other weight loss methods. Additionally, participants are asked to share their thoughts on societal support and resources for individuals who have undergone bariatric surgery. A pilot study was conducted with 20 adult participants from the Al-Qassim Region to assess the feasibility of the study design and data collection tools. The questionnaire, comprising demographic information and participants’ perceptions of weight loss surgery, was deemed clear and concise. No issues arose during data collection, affirming the feasibility of the study design. Based on the pilot study's results, minor modifications were implemented before the main study to enhance accuracy and reliability. The results of the pilot study were omitted from the study sample.

In the main study, data analysis was performed using Statistical Product and Service Solutions (SPSS, version 26) (IBM SPSS Statistics for Windows, Armonk, NY). Categorical variables were presented as frequencies and percentages, and a chi-square test was employed to compare participants' perceptions regarding weight loss surgeries and demographic data. A p-value of < 0.05 indicated statistical significance in the analysis of participants' thoughts about weight loss surgeries and related demographic factors and then further displayed as tables or pie charts, accordingly.

## Results

The participants’ sociodemographic data

**Figure 1:**
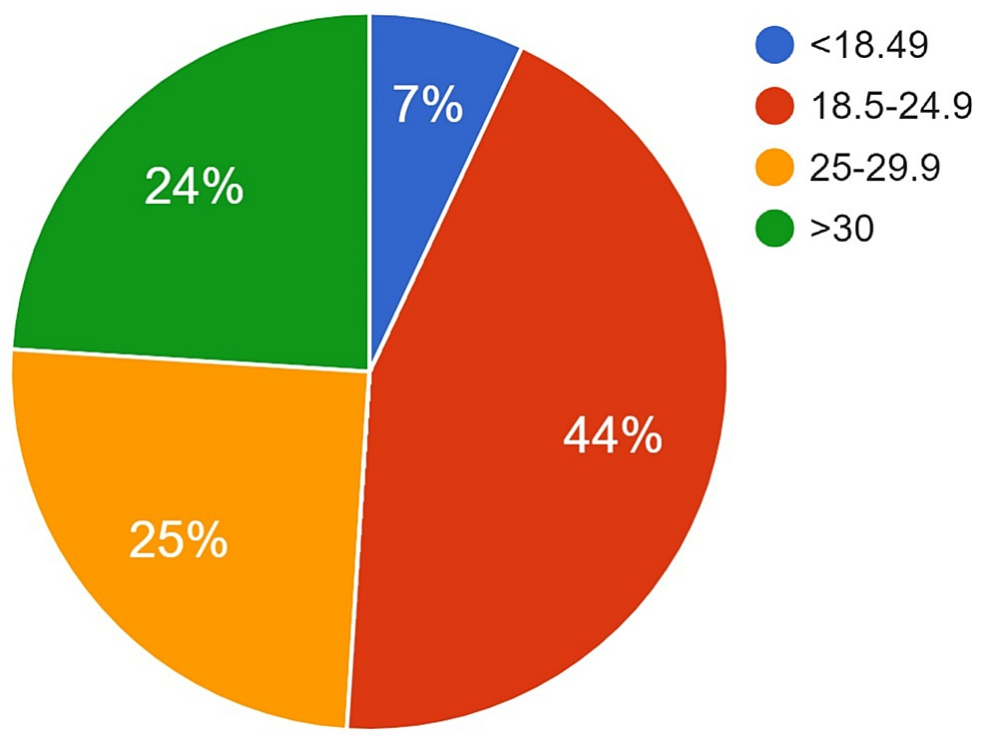
A pie chart depicting the participants as per BMI (n=988)

A total of 988 participants agreed to participate in the study, and their sociodemographic characteristics are presented in Table 1. Specifically, 61.20% (n=605) of the participants were females and had a university education 69.9% (n=691). With respect to age groups (missing 65 values), the largest proportion (43.7%, n=403) of the participants were within the age group of 18-24. With respect to BMI (missing 36 values), the most common category (43.5%, n=414) was 18.5-24.9 (Figure 1).

**Table 1:** Demographic characteristics of the respondents (n=988) *Variables have missing values (65 records in age and 36 records in BMI)

Parameter	Category	N	%
Gender	Male	383	38.80%
	Female	605	61.20%
Educational level	Elementary	6	0.60%
	Middle	35	3.50%
	High (Secondary)	154	15.60%
	University	691	69.90%
	Post-graduate	93	9.40%
	Other	9	0.90%
Age*	18-24	403	43.70%
	25-35	182	19.70%
	36-45	178	19.30%
	46-65	156	16.90%
	≥ 66	4	0.40%
BMI*	< 18.49	71	7.50%
	18.5-24.9	414	43.50%
	25-29.9	236	24.80%
	30 or more	231	24.30%

Knowledge of bariatric surgery

Knowledge about bariatric surgery is presented in Table 2. The majority of the participants (57.30%, n=566) agreed strongly that obesity is a disease, while 30.50% (n=301) agreed, and 9.30% (n=92) remained neutral. With respect to genetic factors’ role in causing obesity, a substantial proportion of them (38.80%, n=383) strongly agreed, and 43.90% (n=434) agreed that genetic factors are one of the causes of obesity. When asked about the factors that increase the risk of obesity, the options selected most commonly were “idle and lazy life” 76.50% (n=756) and “eating too much” 75.60% (n=747). A significant proportion of the participants also indicated other factors that were associated with obesity, such as genetic factors (68.40%, n=676), stress and tension (34.60%, n=342), and psychological diseases (39.00%, n=385). With respect to perceptions about obesity, a notable proportion of them disagreed or strongly disagreed that most obese people are lazy (24.80%, n=245) and that excessive eating is the primary reason that most individuals suffer from obesity (14.00%, n=138). With respect to blame for obesity, a majority of the participants disagreed (28.00%, n=277) and strongly disagreed (17.70%, n=175) that people who are overweight or obese should be blamed for their condition.

**Table 2:** Participants’ responses regarding their knowledge about obesity (n=988)

Parameter	Category	N	%
Obesity is a disease	Strongly disagree	2	0.20%
	Disagree	27	2.70%
	Neutral	92	9.30%
	Agree	301	30.50%
	Strongly agree	566	57.30%
Genetic factors are one of the causes of obesity	Strongly disagree	5	0.50%
	Disagree	38	3.80%
	Neutral	128	13.00%
	Agree	434	43.90%
	Strongly agree	383	38.80%
Which of the following increases the risk of obesity?	Genetic	676	68.40%
	Idle and lazy life	756	76.50%
	Too much eating	747	75.60%
	Disease (of all kinds)	412	41.70%
	Stress and tension	342	34.60%
	Psychological diseases	385	39.00%
	Other	14	1.40%
Most obese people are lazy	Strongly disagree	35	3.50%
	Disagree	210	21.30%
	Neutral	289	29.30%
	Agree	265	26.80%
	Strongly agree	189	19.10%
Excessive eating is the main reason for most of those who suffer from obesity	Strongly disagree	13	1.30%
	Disagree	125	12.70%
	Neutral	164	16.60%
	Agree	415	42.00%
	Strongly agree	271	27.40%
People who are overweight or obese should be blamed for their condition	Strongly disagree	175	17.70%
	Disagree	277	28.00%
	Neutral	232	23.50%
	Agree	182	18.40%
	Strongly agree	122	12.30%
Choose the tips that you think should be directed to people who are overweight or obese	Reducing the amounts of food	690	69.80%
	Prevent sugars	523	52.90%
	If you only exercise, you will lose weight	324	32.80%
	An operation to get rid of obesity	187	18.90%
	Take diet pills	81	8.20%
	You must have the motivation and will	663	67.10%
	Reduce sleep because more sleep increases weight	92	9.30%
	I don't suggest any of these tips	57	5.80%
Have you ever thought about treating obesity through surgical operations?	No	439	44.40%
	Yes	95	9.60%
	I have undergone the operation before	37	3.70%
	No (but I know someone who did the operation)	417	42.20%

**Figure 2:**
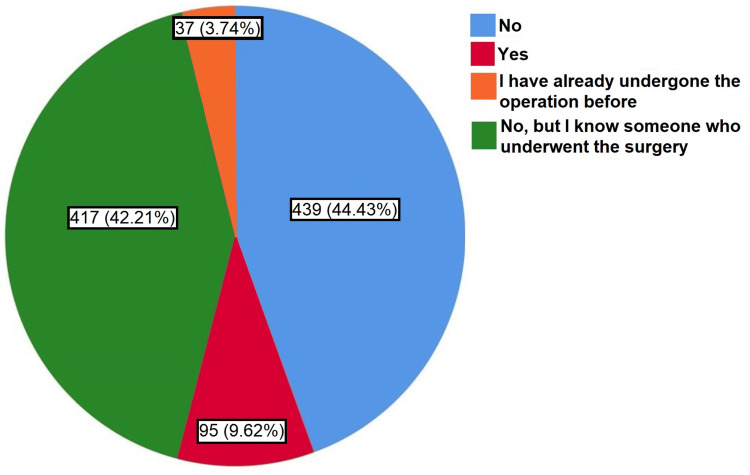
A pie chart depicting the proportions of participants based on having ever thought about treating obesity through surgical operations (n=988)

The participants were also asked to choose the tips that they believe should be offered to individuals who are overweight or obese. The options selected most commonly were “reducing the amounts of food” (69.80%, n=690), having the motivations and will (67.10, n=663), and “prevent sugars” (52.90%, n=523) (Table 2). Importantly, less than half of them (44.43%, n=439), responded that they had never thought about treating obesity through surgical operations, while 9.62% (n=95) had considered it, and 3.74% (n=37) had actually undergone the operation (Figure 2).

The participants’ experience after weight loss surgeries among those who underwent the procedure

The respondents who underwent weight loss surgeries (3.74%, n=37) were asked about their experiences after they underwent the operations, and 43.20% (n=16) of them reported that they faced critical comments or poor treatment from the community because of the bariatric surgery they had. With respect to feeling ashamed or embarrassed to disclose their surgery, 35.10% (n=13) of the participants answered “yes”, while the majority 64.90% (n=24) answered “no.” In addition, 37.80% (n=14) of them reported that they avoid social situations or events because of critical comments or poor treatment received from the community, while 62.20% (n=23) stated that they did not avoid such situations. They were also asked about specific comments that they had received about their surgery. The comments reported most commonly were “You have taken the easy way out instead of adopting a healthy lifestyle,” and “Why didn’t you try to go on a diet?” (51.40%, n=19) of the respondents reported both. Other comments mentioned frequently included “Why didn’t you try exercising instead of surgery?” (43.20%, n=16) and “After the operation, a lot of loose skin will result” (48.60%, n=18). It is worth noting that 21.60% (n=8) of the participants reported that they had not received any critical comments. With respect to the support and resources that would be helpful for individuals who have undergone bariatric surgery, the majority of them 48.60% (n=18) mentioned the importance of receiving advice and access to community educational sources or resources. (45.90%, n=17) of the respondents also considered that support groups that consisted of individuals who underwent the same experience were beneficial. Volunteer campaigns and “other” resources were mentioned by smaller proportions of the participants (32.40%, n=12, and 2.70%, n=1, respectively). Further, they were asked about their opinions on the effect of increasing public awareness and education about obesity treatment and its benefits in reducing misconceptions. A significant majority (62.20%, n=23) strongly agreed that increasing public awareness and education can help reduce inaccurate impressions, while 21.60% (n=8) agreed, and 10.80% (n=4) remained neutral (Table 3).

**Table 3:** Participants’ experience after weight loss surgeries among those who underwent surgeries (n=37)

Parameter	Category	N	%
Have you been exposed to negative comments or bad treatment from the community because of the bariatric surgery that you performed?	No	21	56.80%
	Yes	16	43.20%
Do you feel ashamed or embarrassed to tell others that you have undergone bariatric surgery?	No	24	64.90%
	Yes	13	35.10%
Have you ever avoided social situations or events because of the negative comments or bad treatment from the community that were directed at you because of the bariatric surgery that you had?	No	23	62.20%
	Yes	14	37.80%
Have you been subjected to comments such as:	You have taken the easy way out instead of adopting a healthy lifestyle	19	51.40%
	don't you go on a diet?	19	51.40%
	Why didn't you try exercising instead of surgery?	16	43.20%
	After the operation, a lot of loose skin will result	18	48.60%
	I have not received any negative comments	8	21.60%
	You can live with obesity without resorting to operations	10	27.00%
	Certainly, there were other solutions to obesity, such as (sports, intense diet....etc.).	11	29.70%
Those around you were motivating you to have the operation?	Strongly disagree	1	2.70%
	Disagree	1	2.70%
	Neutral	13	35.10%
	Agree	12	32.40%
	Strongly agree	10	27.00%
What kind of support or resources do you think would be helpful for individuals who have undergone bariatric surgery to deal with any negative experiences or treatment from the community?	Volunteer campaigns	12	32.40%
	Advice	18	48.60%
	Community educational sources or resources	18	48.60%
	Support groups (those who underwent the same experience)	17	45.90%
	Other	1	2.70%
Do you think that increasing public awareness and education about obesity treatment and its benefits can reduce the wrong impression?	Strongly disagree	1	2.70%
	Disagree	1	2.70%
	Neutral	4	10.80%
	Agree	8	21.60%
	Strongly agree	23	62.20%

Intentions to undergo weight loss surgery

The participants’ intentions to undergo weight loss surgery and their concerns related to public opinion and community treatment were assessed (Table 4). A significant proportion of the participants disagreed (30.1%, n=28), and slightly fewer strongly agreed (25.80%, n=24) that concerns about public opinion or community treatment could affect their decision to undergo surgery. Moreover, 41.10% (n=39) of the participants disagreed, while 17.90% (n=17) strongly agreed that society holds a negative attitude toward individuals who have undergone obesity treatment. When asked whether they had ever avoided telling people that they were considering the surgery because of potential critical reactions, 42.10% (n=40) of them responded affirmatively, while the majority (57.90%, n=55) indicated that they had not avoided disclosing this information.

**Table 4:** Intentions to undergo weight loss surgery (n=95) *The variable had two missing values.

Parameter	Category	N	%
Do concerns about public opinion or community treatment affect your decision to take the operation as a treatment for obesity?*	Strongly disagree	10	10.80%
	Disagree	28	30.10%
	Neutral	15	16.10%
	Agree	16	17.20%
	Strongly agree	24	25.80%
Society takes a negative attitude toward individuals who undergo surgery to treat obesity.	Strongly disagree	6	6.30%
	Disagree	39	41.10%
	Neutral	19	20.00%
	Agree	14	14.70%
	Strongly agree	17	17.90%
Have you ever avoided telling people that you are considering surgery because of potential negative reactions?	No	55	57.90%
	Yes	40	42.10%
Those around you were motivating you to have the operation.	Strongly disagree	8	8.40%
	Disagree	17	17.90%
	Neutral	35	36.80%
	Agree	26	27.40%
	Strongly agree	9	9.50%
Public opinion can make the decision about taking the operation to treat obesity difficult.	Strongly disagree	3	3.20%
	Disagree	20	21.10%
	Neutral	24	25.30%
	Agree	28	29.50%
	Strongly agree	20	21.10%
How does public opinion about bariatric surgery affect individuals who are considering it?	Fear of negative reactions from others	44	46.30%
	Fear of discrimination and prejudice	24	25.30%
	Low self-esteem and self-confidence	30	31.60%
	Other	49	51.60%

The participants were also asked whether those around them had motivated them to have the surgery. The majority (36.80%, n=35) responded neutrally, while 27.40% (n=26) agreed, and 9.50% (n=9) strongly agreed that those around them had motivated them to undergo weight loss surgery. With respect to public opinion’s effect on their decision to undergo surgery, 29.50% (n=28) of the participants agreed, and 21.10% (n=20) strongly agreed that public opinion can make the decision about having the operation to treat obesity difficult. The study also investigated the way that public opinion about bariatric surgery affects individuals who are considering it. The effects reported most commonly were fear of others’ critical reactions (46.30%, n=44) and low self-esteem and self-confidence (31.60%, n=30). The majority of the participants answered neutral (36.80%, n=35) with respect to whether those around them were motivating them to have the operation, followed by agreement with this statement (27.40%, n=26) (Table 4).

Perceptions with respect to operations to treat obesity

The participants’ perceptions of operations to treat obesity were assessed through various statements. With respect to the belief that most individuals who underwent the operation as a treatment for obesity failed to follow a healthy diet and exercise thereafter, the majority of the participants (32.20%, n=318) responded neutrally, followed by 30.20% (n=298) who agreed with the statement. When asked about the perception that most bariatric surgeries are limited only to lazy individuals, a significant proportion of the participants (38.70%, n=382) disagreed, while 12.00% (n=119) strongly agreed with the statement. With respect to the belief that people who had bariatric surgery had other weight loss options available to them, 42.30% (n=418) agreed, and 21.00% (n=207) strongly agreed. With respect to the perception that bariatric surgery is the easiest way to lose weight compared to making efforts to follow a diet and exercise, 29.00% (n=287) agreed, while 21.60% (n=213) strongly agreed. When asked about the appropriateness for young men and young women to undergo obesity surgery, the majority of the participants (79.00%, n=781) believed that it was inappropriate except in severe cases, while 7.90% (n=78) thought that it was unsuitable in all cases.

A majority of the participants (47.40%, n=468) agreed, and 24.10% (n=238) strongly agreed and acknowledged the importance of addressing public opinion of bariatric surgery, while a smaller proportion disagreed or strongly disagreed (5.50%, n=54). The participants’ views on whether bariatric surgery is cosmetic surgery varied, with 33.90% (n=335) disagreeing, 26.90% (n=266) responding neutrally, and 20.30% (n=201) agreeing with the statement. With respect to the perception of the quality of life of obese people who underwent bariatric surgery, a similar proportion of the participants agreed (32.90%, n=325) and answered neutral (32.60%, n=322), while 17.60% (n=174) strongly agreed with the statement. The majority of them (37.10%, n=367) agreed that those who underwent bariatric surgery should be congratulated. With respect to the belief that health insurance should cover the cost of the operation to facilitate access, 35.30% (n=349) agreed, 30.20% (n=298) strongly agreed, and 9.20% (n=91) disagreed or strongly disagreed (Table 5).

**Table 5:** Participants’ responses to their perceptions regarding operations to treat obesity (n=988)

Most of those who underwent the operation as a treatment for obesity failed to follow a healthy diet and exercise	Strongly disagree	24	2.40%
	Disagree	178	18.00%
	Neutral	318	32.20%
	Agree	298	30.20%
	Strongly agree	170	17.20%
Most bariatric surgeries are only limited to lazy individuals	Strongly disagree	106	10.70%
	Disagree	382	38.70%
	Neutral	217	22.00%
	Agree	164	16.60%
	Strongly agree	119	12.00%
People who had bariatric surgery had other options available to them to lose weight.	Strongly disagree	17	1.70%
	Disagree	93	9.40%
	Neutral	253	25.60%
	Agree	418	42.30%
	Strongly agree	207	21.00%
Bariatric surgery is the easiest way to lose weight instead of making efforts to follow a diet and exercise	Strongly disagree	96	9.70%
	Disagree	214	21.70%
	Neutral	178	18.00%
	Agree	287	29.00%
	Strongly agree	213	21.60%
It is wrong for young men/young women to undergo obesity surgery	No	129	13.10%
	Yes (except for severe cases)	781	79.00%
	Yes (in all cases)	78	7.90%
It is important to address public opinion regarding bariatric surgery	Strongly disagree	11	1.10%
	Disagree	43	4.40%
	Neutral	228	23.10%
	Agree	468	47.40%
	Strongly agree	238	24.10%
Bariatric surgery is a cosmetic surgery	Strongly disagree	104	10.50%
	Disagree	335	33.90%
	Neutral	266	26.90%
	Agree	201	20.30%
	Strongly agree	82	8.30%
The quality of life of obese people who underwent bariatric surgery is likely to be better than that of obese people who did not undergo the operation.	Strongly disagree	37	3.70%
	Disagree	130	13.20%
	Neutral	322	32.60%
	Agree	325	32.90%
	Strongly agree	174	17.60%
Those who underwent bariatric surgery should be congratulated	Strongly disagree	21	2.10%
	Disagree	94	9.50%
	Neutral	348	35.20%
	Agree	367	37.10%
	Strongly agree	158	16.00%
The health insurance must cover the cost of the operation in order to facilitate access to it	Strongly disagree	18	1.80%
	Disagree	73	7.40%
	Neutral	250	25.30%
	Agree	349	35.30%
	Strongly agree	298	30.20%

Factors associated with the participants’ attitudes and practice toward weight-loss surgeries

Table 6 presents the association between the participants’ demographic characteristics and their attitudes toward weight-loss surgeries. Among the variables examined, only two showed a significant association with the participants’ attitudes. Age was found to be associated significantly with the participants’ thoughts about treating obesity through surgical operations (p<0.001). Specifically, participants aged 46-65 years (7.1%, n=11) and 25-35 years (5.5%, n=10) underwent weight loss surgeries significantly more often compared to other age categories (2.7%, 2.2%, and 0.0% (n=11, 4, 0) among those aged 18-24, 36-45 years, and > 66 years, respectively. Significantly more participants with higher BMI levels, in which 5.2% (n=12) and 5.1% (n=12) were overweight and obese, respectively, had undergone surgery compared to other categories (3.1%, n=13; among a normal BMI and 0.0% among underweight; p<0.0001; Table 6).

**Table 6:** The association between demographic characteristics and participants’ attitudes towards weight-loss surgeries p<0.05 is considered statistically significant.

Parameter	Category	Have you ever thought about treating obesity through surgical operations?								P value
		No		Yes		No (but I know someone who did the operation)		I have undergone the operation before		
		N	%	N	%	N	%	N	%	
Gender	Male	184	48.00%	40	10.44%	144	37.60%	15	3.92%	0.140
	Female	255	42.10%	55	9.09%	273	45.12%	22	3.64%	
Educational level	Elementary	4	66.7%	2	33.3%	0	0.0%	0	0.0%	0.432
	Middle	11	31.4%	4	11.4%	18	51.4%	2	5.7%	
	High (Secondary)	69	44.8%	12	7.8%	68	44.2%	5	3.2%	
	University	306	44.3%	65	9.4%	295	42.7%	25	3.6%	
	Post-graduate	46	49.5%	10	10.8%	33	35.5%	4	4.3%	
	Other	3	33.3%	2	22.2%	3	33.3%	1	11.1%	
Age	18-24	170	42.2%	26	6.5%	196	48.6%	11	2.7%	< 0.001
	25-35	78	42.9%	21	11.5%	73	40.1%	10	5.5%	
	36-45	90	50.6%	27	15.2%	57	32.0%	4	2.2%	
	46-65	80	51.3%	18	11.5%	47	30.1%	11	7.1%	
	≥ 66	3	75.0%	0	0.0%	1	25.0%	0	0.0%	
BMI	< 18.49	28	39.4%	2	2.8%	41	57.7%	0	0.0%	< 0.001
	18.5-24.9	180	43.5%	14	3.4%	207	50.0%	13	3.1%	
	25-29.9	107	45.3%	18	7.6%	99	41.9%	12	5.1%	
	30 or more	98	42.4%	61	26.4%	60	26.0%	12	5.2%	

The association between the participants’ perception of the operation to treat obesity and their attitudes toward weight-loss surgeries

Table 7 presents the association between the participants’ perception of the operation to treat obesity and their attitudes toward weight-loss surgeries. The highest percentage (20.8%, n=5) of the participants strongly disagreed with the statement, “Most of those who underwent the operation as a treatment for obesity failed to follow a healthy diet and exercise” had undergone the operation before (p<0.001). Similarly, the highest percentage (4.7%, n=5) of the participants who strongly disagreed with the statement “Most bariatric surgeries are only limited to lazy individuals” had undergone the operation before (p<0.001). In addition, the highest percentage (14.0%, n=18) of the participants who disagreed with the statement “It is wrong for young men/young women to undergo obesity surgery” had undergone the operation before (p<0.001). Moreover, the highest percentage (5.8%, n=6) of the participants who strongly disagreed with the statement “Bariatric surgery is a cosmetic surgery” had undergone the operation before (p<0.001). In contrast, the lowest percentage of the participants (0.0%, n=0) who strongly disagreed with the statement “Bariatric surgery is the easiest way to lose weight instead of making efforts to follow a diet and exercise” had undergone the operation before (p<0.001). Similarly, the lowest percentage (0.0%, n=0) of the participants who strongly disagreed with the statement “It is important to address public opinion with respect to bariatric surgery” had undergone the operation before (p<0.001) and the lowest percentage (0.0%, n=0) of the participants who strongly disagreed with the statement “The quality of life of obese people who underwent bariatric surgery is likely to be better than that of obese people who did not undergo the operation” had undergone the operation before (p<0.001, Table 7).

**Table 7:** The association between participants’ perception regarding the operations to treat obesity and participants’ attitudes towards weight-loss surgeries P < 0.05 is considered statistically significant.

Parameter	Category	Have you ever thought about treating obesity through surgical operations?								P value
		No		Yes		No (but I know someone who did the operation)		I have undergone the operation before		
		N	%	N	%	N	%	N	%	
Most of those who underwent the operation as a treatment for obesity failed to follow a healthy diet and exercise	Strongly disagree	5	20.8%	3	12.5%	11	45.8%	5	20.8%	< 0.001
	Disagree	67	37.6%	16	9.0%	88	49.4%	7	3.9%	
	Neutral	148	46.5%	22	6.9%	138	43.4%	10	3.1%	
	Agree	128	43.0%	29	9.7%	134	45.0%	7	2.3%	
	Strongly agree	91	53.5%	25	14.7%	46	27.1%	8	4.7%	
Most bariatric surgeries are only limited to lazy individuals	Strongly disagree	32	30.2%	10	9.4%	59	55.7%	5	4.7%	< 0.001
	Disagree	151	39.5%	37	9.7%	181	47.4%	13	3.4%	
	Neutral	104	47.9%	16	7.4%	90	41.5%	7	3.2%	
	Agree	78	47.6%	12	7.3%	67	40.9%	7	4.3%	
	Strongly agree	74	62.2%	20	16.8%	20	16.8%	5	4.2%	
People who had bariatric surgery had other options available to them to lose weight.	Strongly disagree	7	41.2%	3	17.6%	4	23.5%	3	17.6%	0.001
	Disagree	31	33.3%	5	5.4%	49	52.7%	8	8.6%	
	Neutral	111	43.9%	28	11.1%	102	40.3%	12	4.7%	
	Agree	186	44.5%	38	9.1%	185	44.3%	9	2.2%	
	Strongly agree	104	50.2%	21	10.1%	77	37.2%	5	2.4%	
Bariatric surgery is the easiest way to lose weight instead of making efforts to follow a diet and exercise	Strongly disagree	47	49.00%	1	1.00%	48	50.00%	0	0.00%	< 0.001
	Disagree	91	42.50%	12	5.60%	105	49.10%	6	2.80%	
	Neutral	95	53.40%	13	7.30%	63	35.40%	7	3.90%	
	Agree	119	41.50%	32	11.10%	124	43.20%	12	4.20%	
	Strongly agree	87	40.80%	37	17.40%	77	36.20%	12	5.60%	
It is wrong for young men/young women to undergo obesity surgery	No	51	39.50%	17	13.20%	43	33.30%	18	14.00%	< 0.001
	Yes (except for severe cases)	340	43.50%	67	8.60%	358	45.80%	16	2.00%	
	Yes (in all cases)	48	61.50%	11	14.10%	16	20.50%	3	3.80%	
It is important to address public opinion regarding bariatric surgery	Strongly disagree	8	72.7%	2	18.2%	1	9.1%	0	0.0%	< 0.001
	Disagree	21	48.8%	3	7.0%	16	37.2%	3	7.0%	
	Neutral	120	52.6%	25	11.0%	77	33.8%	6	2.6%	
	Agree	207	44.2%	33	7.1%	215	45.9%	13	2.8%	
	Strongly agree	83	34.9%	32	13.4%	108	45.4%	15	6.3%	
Bariatric surgery is a cosmetic surgery	Strongly disagree	24	23.10%	7	6.70%	67	64.40%	6	5.80%	< 0.001
	Disagree	143	42.70%	32	9.60%	150	44.80%	10	3.00%	
	Neutral	131	49.20%	23	8.60%	103	38.70%	9	3.40%	
	Agree	99	49.30%	23	11.40%	71	35.30%	8	4.00%	
	Strongly agree	42	51.20%	10	12.20%	26	31.70%	4	4.90%	
The quality of life of obese people who underwent bariatric surgery is likely to be better than that of obese people who did not undergo the operation.	Strongly disagree	15	40.50%	0	0.00%	22	59.50%	0	0.00%	< 0.001
	Disagree	67	51.50%	4	3.10%	58	44.60%	1	0.80%	
	Neutral	168	52.20%	23	7.10%	124	38.50%	7	2.20%	
	Agree	124	38.20%	37	11.40%	155	47.70%	9	2.80%	
	Strongly agree	65	37.40%	31	17.80%	58	33.30%	20	11.50%	
Those who underwent bariatric surgery should be congratulated	Strongly disagree	11	52.4%	0	0.0%	10	47.6%	0	0.0%	< 0.001
	Disagree	38	40.4%	4	4.3%	50	53.2%	2	2.1%	
	Neutral	185	53.2%	21	6.0%	135	38.8%	7	2.0%	
	Agree	155	42.2%	36	9.8%	165	45.0%	11	3.0%	
	Strongly agree	50	31.6%	34	21.5%	57	36.1%	17	10.8%	
The health insurance must cover the cost of the operation in order to facilitate access to it	Strongly disagree	8	44.4%	1	5.6%	9	50.0%	0	0.0%	< 0.001
	Disagree	29	39.7%	4	5.5%	37	50.7%	3	4.1%	
	Neutral	144	57.6%	10	4.0%	92	36.8%	4	1.6%	
	Agree	150	43.0%	33	9.5%	162	46.4%	4	1.1%	
	Strongly agree	108	36.2%	47	15.8%	117	39.3%	26	8.7%	

The association between the participants’ perception of the operation to treat obesity and sociodemographic data

Tables 8-9 present the association between the participants’ perception of the operation to treat obesity and sociodemographic data. A statistically significant difference was observed in gender (p<0.001). The median value of the parameter for males was found to be 32.0 (IQR: 29.0-35.0), which was higher than for females. The participants were categorized based on their educational level, and the median values were higher in middle school (31.0, IQR: 29.0-35.0), university (31.0, IQR: 27.0-34.0), and post-graduate (31.0, IQR: 29.0-35.0). A statistically significant difference was observed among the educational levels (p=0.016). According to age groups (p<0.001), the median value for the age group ≥ 66 was 38.0 (IQR: 34.0-43.5) and was the highest median among the age groups. With respect to BMI categories, the median value for category 30 or more was 33.0 (IQR: 28.0-35.0), and there was a statistically significant difference among the BMI categories (p<0.001). The effect of the male participants’ perception of the operations to treat obesity was significantly stronger compared to that of females (beta=1.910, 95% CI: 1.290-2.531, p<0.001). The participants in the age groups 18-24 (beta=-7.563, 95% CI: -11.985 to -3.141, p=0.001), 25-35 (beta=-5.005, 95% CI: -9.442 to -0.569, p=0.027), 36-45 (beta=-5.646, 95% CI: -10.079 to -1.212, p=0.013), and 46-65 (beta=-5.642, 95% CI: -10.075 to -1.209, p=0.013) showed a significantly weaker effect on the perception of the operations to treat obesity compared to the reference group (≥66 years). The participants with a BMI of ≤18.49 (beta=-2.139, 95% CI: -3.513 to -0.765, p=0.002), 18.5-24.9 (beta=-1.142, 95% CI: -1.937 to -0.346, p=0.005), and 25-29.9 (beta=-1.437, 95% CI: -2.262 to -0.613, p=0.001) exhibited a significantly lower effect on the parameter compared to those with a BMI of 30 or more (Tables 8-9).

**Table 8:** The association between participants’ perception regarding the operations to treat obesity and sociodemographic data p<0.05 is considered statistically significant.

Parameter	Category	Median (IQR)	P value
Gender	Male	32.0 (29.0-35.0)	< 0.001
	Female	30.0 (27.0-33.0)	
Educational level	Elementary	27.5 (26.0-32.5)	0.016
	Middle	31.0 (29.0-35.0)	
	High	30.0 (26.75-33.0)	
	University	31.0 (27.0-34.0)	
	Post-graduate	31.0 (29.0-35.0)	
	Other	29.0 (26.0-38.0)	
Age	18-24	29.0 (26.0-32.0)	< 0.001
	25-35	32.0 (29.0-35.0)	
	36-45	31.0 (28.0-34.0)	
	46-65	32.0 (29.0-35.0)	
	≥ 66	38.0 (34.0-43.5)	
BMI	≤ 18.49	29.0 (26.0-32.0)	< 0.001
	18.5-24.9	30.0 (27.0-33.0)	
	25-29.9	31.0 (28.0-34.0)	
	30 or more	33.0 (28.0-35.0)	

**Table 9:** The relation between participants’ perception regarding the operations to treat obesity and sociodemographic data (regression analysis) p<0.05 is considered statistically significant. CI = Confidence Interval, LB = Lower Bound, UB = Upper Bound, Ref = Reference

Parameter	Category	Beta	95% CI		P value
			LB	UB	
Gender	Male	1.910	1.290	2.531	< 0.001
	Female	Ref.	Ref.	Ref.	Ref.
Educational level	Elementary	-2.859	-7.548	1.831	0.232
	Middle	0.438	-3.435	4.31	0.824
	High	-0.151	-3.376	3.074	0.927
	University	-0.363	-3.494	2.768	0.820
	Post-graduate	0.048	-3.181	3.278	0.977
	Other	Ref.	Ref.	Ref.	Ref.
Age	18-24	-7.563	-11.985	-3.141	0.001
	25-35	-5.005	-9.442	-0.569	0.027
	36-45	-5.646	-10.079	-1.212	0.013
	46-65	-5.642	-10.075	-1.209	0.013
	≥ 66	Ref.	Ref.	Ref.	Ref.
BMI	≤ 18.49	-2.139	-3.513	-0.765	0.002
	18.5-24.9	-1.142	-1.937	-0.346	0.005
	25-29.9	-1.437	-2.262	-0.613	0.001
	30 or more	Ref.	Ref.	Ref.	Ref.

## Discussion

Obesity is a major public health concern that is associated with numerous health problems, including diabetes, heart disease, and certain types of cancer [11]. Locally, obesity in Saudi Arabia is sitting at 24%; however, when compared to previous years, this number is lower [12]. As for why this is happening, there is no justified answer as of yet, but there are some speculations that it may be as a result of policies, which encourage a healthier lifestyle [12]. Although the use of bariatric surgery is only one of the options available to manage obesity and associated illnesses, it remains the most effective [8]. However, the pervasive stigma associated with bariatric surgery may limit the candidate’s willingness to undergo the operation and have an effect on patients’ well-being both before and after the surgery. Therefore, it is crucial to carry out a study on these stigma’s nature and effects.

To our knowledge, no single study in Saudi Arabia has been conducted to assess the effect of public stigmatization related to bariatric surgeries on both those who have undergone the surgery and those who are considering it as an option. Further, in our study, we assessed the public’s knowledge, attitude, and prevalence of obesity among males/females in the Al-Qassim Region.

The participants’ sociodemographic data

In this study of Saudi adults in the Al-Qassim Region, the BMI of nearly 43.50% (n=414) of the respondents was within the normal range, while 49.1% (n=467) were classified as overweight or obese. This prevalence is consistent with that of another study in the region that found that the prevalence of obesity and being overweight was 53.5% [13]. Further, it is consistently compared to other studies that have shown that the prevalence of obesity and being overweight were 57.7%, 40%, and 54%, respectively, among adults across Saudi Arabia [14-16].

Knowledge about bariatric surgery

In this study, we observed that the participants had an appropriate level of knowledge about obesity. For example, 87.8% (n=867) of them strongly agreed or agreed that obesity is a disease, and 82.7% (n=817) believed that genetic factors may play a large role in obesity. Additionally, 76.50% (n=756) of them believe that the factors of an idle and lazy life, eating too much (75.60%, n=747), disease of all kinds (41.70%, n=412), stress and tension (34.60%, n=342), and psychological diseases (39.00%, n=385) can increase the risk of obesity. In comparison, our finding is consistent with those in local studies in Riyadh City published in January 2018 and in the Al-Qassim Region published in February 2019, in which the authors found that most of the respondents have a high level of knowledge about obesity’s causes and risk factors [13,16]. Another national study showed that the stereotype perceived most commonly was that obese individuals are lazy, which 62% of their sample endorsed [17]. In our study, with respect to blame for obesity, a majority of the participants disagreed (28.00%, n=277) and strongly disagreed (17.70%, n=175) that people who are overweight or obese should be blamed for their condition. In contrast to another study in which the participants were asked whether others had ever blamed them for their weight problems, the majority (66.4%) responded that they had not by any means [18]. Importantly, less than half of the participants (44.43%, n=439) responded that they had never considered treating obesity through surgical operations, 9.62% (n=95) had considered it, and 3.74% (n=37) had actually undergone the operation.

The participants’ experience after weight-loss surgeries

In this study, the patients who underwent bariatric surgery (3.74%, n=37) were asked about the weight stigma and their experiences after the surgery. Moreover, 43.20% (n=16) stated that they were exposed to unpleasant comments or poor treatment from the community, and 35.1% (n=13) felt ashamed or embarrassed to disclose their surgery. “You have taken the easy way out instead of adopting a healthy lifestyle” and “Why didn’t you try to go on a diet?” were the most common comments that they received from the community (51.4%, n=19). This result is consistent with that of another study conducted in Brazil [9]. Further, in this study, most of the participants agreed about the importance of receiving advice and access to community educational sources or resources (48.6%, n=18) to support those who had undergone the surgery. This is consistent with the evidence in a study conducted to investigate social support’s effect on mental health and eating disorders after the surgery was performed and showed that greater social support was associated with lower depression [19].

Intentions to undergo weight-loss surgery

When we investigated what may affect a patient’s decision, their intention whether or not to opt for weight-loss surgery, and their concerns about the matter pre- or post-surgery, we found in our demographic survey that public concern with respect to the surgery had an overall mixed opinion, indicating that it may not be necessary to consider it. In addition, it was seen that society’s view toward those who did undergo the surgery does not seem to be critical overall. As per the other points assessed as well, such as whether some had avoided telling others that they underwent the surgery, or whether those around them played a part in their decision to have it or not, nearly equal numbers tended to be neutral. However, the majority of the participants agreed that the public’s perception of the surgery may make it more difficult for someone to undergo the surgery, albeit they said earlier that public opinion was not much of an issue. When considering if public opinion had affected an individual’s opinion regarding undergoing the surgery, "fear of negative reactions from others" had been predominantly chosen, followed by "low self-esteem and self-confidence," similarly pointed out in a systemic review, in a qualitative study, that people may try to hide their weight loss or the fact that they have undergone surgery as a means of avoiding the stigma and negative comments, which may be directed towards them [20].

Perceptions of surgery to treat obesity

In our study, we found that the majority of the participants tended to believe that individuals who had undergone bariatric surgery to treat obesity had failed to follow a healthy diet and exercise subsequently. This was confirmed further when, again, the majority of them said that those who underwent the procedure had other options available to lose weight and that surgery was the easiest way, which was consistent with the results of other studies [10,21,22].

While there were mixed opinions in our study when the participants were asked whether bariatric surgery was considered cosmetic, rather than therapeutic, one of the largest studies related to bariatric surgery’s effect on obesity and its outcomes, the Swedish Obesity Study, has concluded that "bariatric surgery for severe obesity is associated with long-term weight loss and decreased overall mortality," which emphasizes the therapeutic side of the surgery. The study compared groups of patients who had undergone the surgical procedure to control their weight change/loss. Moreover, the group who had undergone the procedure for weight loss had a far more positive outcome and/or change in weight loss and any obesity-related outcomes, with the added bonus that it even helped those who had diabetes control their disease better [23].

Nevertheless, our sample also showed that many agreed that the quality of life of those who do undergo the surgery is more likely to be better than those who have not and deemed that health insurance should still cover the procedure’s cost.

Factors associated with the participants’ attitudes and practice toward weight-loss surgeries

It was also noticed that, when we investigated whether there were any factors that affected the way that the surgery was viewed, two variables had a noticeable effect on their attitude and practice toward bariatric surgery. Those variables were age and BMI and showed that individuals who were older or had a higher BMI were more likely to have undergone a weight-loss procedure already. Unexpectedly, gender did not cause a significant change or effect.

The association between the participants’ perception of the operations to treat obesity and their attitudes toward weight-loss surgeries

We examined the association between the participants’ perceptions of operations to treat obesity and their attitudes toward these surgeries in Saudi Arabia. The participants who strongly disagreed with negative statements about bariatric surgery (e.g., being lazy, taking the easy way out, or your quality life would be better if you did not undergo the surgery) had a significantly higher number of prior surgical experiences, indicating a positive correlation between favorable attitudes toward surgery and prior surgical history. These findings are consistent with those in previous research conducted among the Saudi adult population, in which a subset of individuals showed more positive attitudes toward bariatric surgery although the majority preferred a proper diet for weight loss [13]. This suggests that individuals who reject the prevailing negative perceptions and stereotypes associated with weight-loss surgeries may be more inclined to consider bariatric surgery as a viable option to address morbid obesity. These results emphasize the potential effect of efforts to reduce stigma in promoting informed decision-making and increasing eligible candidates’ uptake of bariatric surgery. These findings are reinforced when the strong desire of individuals who have undergone the surgery to increase public awareness and address the stigma associated with this procedure, which had the lowest disagreement 0.0% (n=0), among them (p<0.001), is taken into consideration.

In addition, our study revealed that the participants who undergo the operation face surgery-related stigma, which is supported by a study conducted in a bariatric clinic in the American Southwest that found that people who have bariatric surgery for weight loss may trade one type of stigma for another [24]. Thus, individuals who qualify for bariatric surgery based on weight alone may be reluctant to explore the surgery as a viable option.

Further, these findings are consistent with those in another study that emphasized the importance of addressing weight- and surgery-related stigma at a larger level, stating that societal perceptions’ effect extends beyond individuals who are considering bariatric surgery; it also affects patients’ experiences post-surgery [25]. Our findings supported this further by showing that the participants with positive attitudes toward bariatric surgery were more likely to have undergone the operation [13,25]. This highlights the need for healthcare providers to create a supportive and non-stigmatizing environment to enhance post-surgical outcomes.

The association between the participants’ perception of the operations to treat obesity and sociodemographic data

The significant association between the participants’ perception of obesity treatment operations and various sociodemographic factors, particularly gender, sheds light on the potential gender-specific disparities in attitudes toward weight-loss surgeries. Our study revealed that the male participants’ attitudes had a greater effect on their perception compared to females, suggesting a distinct perspective or awareness with respect to such surgical interventions. However, Aly et al.’s previous study, which explored demographic and quality of life factors that influence patients’ consideration of bariatric surgery reported that a higher proportion of women (40%) had considered weight-loss surgery compared to men (22%), and women’s physicians were more likely to recommend the surgery (22% vs. 14%) [26]. These gender-based disparities indicate that gender-specific societal norms and expectations may influence perceptions and motivations related to weight-loss surgeries. Understanding such nuances is crucial for healthcare providers to develop tailored approaches to support patients’ decision-making processes and address potential barriers to seeking weight-loss surgeries. By recognizing the effect of sociodemographic factors, including gender, on attitudes toward bariatric surgery, healthcare professionals can promote more equitable access to, and use of, this effective treatment option for obesity.

Our findings revealed a significant association between the participants’ perceptions of obesity treatment operations and their educational level. Notably, the participants with higher education, such as university and post-graduate, exhibited similar median values in their perceptions (31.0), suggesting that individuals with more education may possess a better understanding of weight-loss surgeries. Education’s influence on attitudes toward weight-loss surgeries was supported by a separate investigation on laparoscopic sleeve gastrectomy (LSG) treatment. The study implemented three education sessions for one group of patients after LSG, while another control group received only written recommendations in the form of a guidebook. The results demonstrated that receiving education sessions had a significant effect on the study group’s weight loss and adherence to lifestyle recommendations compared to the control group (p<0.001). Moreover, the control group exhibited less motivation to comply with recommendations after a year of observation [27]. These findings underscore education’s importance in promoting successful weight loss and lifestyle changes after bariatric surgery and suggest that integrating education as a permanent element of the LSG procedure can enhance its effectiveness in treating morbid obesity.

Moreover, the findings related to BMI categories showed a statistically significant difference (p<0.001). The participants with a BMI of 30 or more had a higher median value (33.0, IQR: 28.0-35.0) in their perception of obesity treatment operations. This suggests that individuals with a higher BMI may have a different and more positive attitude or opinion toward weight-loss surgeries compared to those with a lower BMI.

Overall, the study highlighted gender, age, educational level, and BMI’s influence on the participants’ perception of obesity treatment operations. These sociodemographic factors should be taken into account when designing educational interventions and public awareness campaigns to improve the population’s understanding and acceptance of weight-loss surgeries.

Limitations

Despite the valuable insights gained from this study, it is important to acknowledge its limitations. Firstly, this research was conducted in the Al-Qassim Region, and, as such, the findings may not be representative of the broader population in Saudi Arabia or other Regions. In addition, the research relied primarily on self-reported data from the participants, which can be subject to recall and social desirability biases. The study’s cross-sectional design provides only a snapshot of attitudes and perceptions at a specific point in time, which makes it impossible to establish causal relationships or track changes over time. Finally, the research focused primarily on the general public’s perspective and did not delve into healthcare professionals’ experiences and perspectives, which could provide a more comprehensive understanding of the issue. These limitations should be taken into account when interpreting the findings and can serve as avenues for further research in the future.

## Conclusions

In conclusion, this study underscored the pressing need to address the stigma and misconceptions associated with bariatric weight loss surgery in the Al-Qassim Region. Societal attitudes’ effect on individuals who have undergone these procedures is evident and leads to feelings of shame and hesitation among those considering such treatments.

Several recommendations emerged from this research to improve these individuals’ quality of life and ensure equitable access to life-changing bariatric surgery. Firstly, comprehensive public awareness campaigns should be launched to dispel myths and misunderstandings about bariatric surgery and educate the public about its benefits and medical necessity for many individuals. Secondly, healthcare professionals must play a pivotal role in this endeavor by engaging in proactive patient education and advocacy, challenging stigmatizing beliefs, and providing resources for those who have undergone the surgery. Moreover, the development of support networks, including support groups and community educational resources, is essential to help individuals navigate the post-surgery journey and combat societal bias’ adverse consequences. By implementing these recommendations, it is possible to create a more informed, empathetic, and supportive environment for individuals considering or recovering from bariatric surgery in the Al-Qassim Region and ultimately improve their physical and mental well-being.

## Appendices

Questionnaire

Demographic Characteristics

Age: _________

Gender:

o   Male

o   Female

Weight (kg):_____________

Height (cm):_____________

Education Level:

o   Elementary

o   Middle School

o   High School

o   College (Non-health Major)

o   College (Health Major)

o   Post-graduate

o   Other:_____

1 - Obesity

Obesity is considered a disease.

○ Strongly Disagree     ○ Disagree      ○ Neutral      ○ Agree      ○ Strongly Agree

Genetic factors are considered one of the causes of obesity.

○ Strongly Disagree     ○ Disagree      ○ Neutral      ○ Agree      ○ Strongly Agree

Which of the following increases the risk of obesity? (Select all that apply).

○ Hereditary     ○  Inactive/Sedentary Lifestyle    ○ Eating too much    ○ Illness (All kinds)   

○ Stress      ○ Psychological Diseases       ○ Other: ______

Most obese people are lazy.

○ Strongly Disagree      ○ Disagree      ○ Neutral      ○ Agree      ○ Strongly Agree

Excessive eating is the main cause of most people suffering from obesity.

○ Strongly Disagree      ○ Disagree       ○ Neutral          ○ Agree            ○ Strongly Agree

People who are overweight or obese should be blamed for their condition.

○ Strongly Disagree      ○ Disagree       ○ Neutral          ○ Agree            ○ Strongly Agree

Choose the advice that you think should be given to people who are overweight or obese.

○  Reducing amount of food     ○  Abstain from sugary food     ○ Surgery to get rid of obesity         

○  Through ONLY exercise, you will lose weight                         ○ Take diet pills

○  Reduce sleep, since excess sleep increases weight             ○ You must have motivation and will

○  I wouldn’t suggest any of these tips                                       ○ Other:______

Have you ever thought about treating obesity through surgical operations?

○ Yes                           ○ No                        ○ No, but I know someone who did

○ I’ve already undergone the procedure          

2 - Experience after the operation

Have you been exposed to negative comments or poor treatment from society because of the obesity surgery you underwent?

○ Yes                           ○ No

If yes, what was your experience? (Optional)

______________________________________

Did you feel ashamed or embarrassed to tell others that you underwent obesity surgery?

○ Yes                           ○ No

Have you ever avoided social situations or events because of negative comments or bad treatment from the society that was directed at you because of your obesity surgery?

○ Yes                           ○ No

Have you been subjected to comments, such as:

○ I took the easy way out instead of adopting a healthy lifestyle.

○ Why don't you go on a diet?

○ Why didn't you try exercising instead of surgery?

○ After the procedure, a lot of sagging skin will result.

○ I have not been subjected to any negative comments.

○ You can live with obesity without resorting to operations.

○ Certainly there were other solutions to obesity, such as (exercise, extreme dieting, etc.).

○ Other: ______

People around you motivated you to have the operation.

○ Strongly Disagree      ○ Disagree       ○ Neutral          ○ Agree            ○ Strongly Agree

What type of support or resources do you think would be helpful for individuals who have undergone bariatric surgery to deal with any negative experiences or treatment from society?

○ Volunteer campaigns

○ Advice

○ Resources or educational resources for the community

○ Support groups (for those who have undergone the same experience)

○ Other: _________

Do you think that increasing public awareness and education about the procedure to treat obesity and its benefits can reduce the wrong impression?

○ Strongly Disagree      ○ Disagree       ○ Neutral          ○ Agree            ○ Strongly Agree

3  - Thinking of undergoing the surgery

Concerns about public opinion or community treatment affect your decision to undergo surgery as a treatment for obesity.

○ Strongly Disagree      ○ Disagree       ○ Neutral          ○ Agree             ○ Strongly Agree

Society takes a negative attitude towards individuals who have undergone obesity treatment surgery.

○ Strongly Disagree      ○ Disagree       ○ Neutral          ○ Agree             ○ Strongly Agree

Have you ever avoided telling people that you are considering surgery because of potential negative reactions?

○ Yes               ○ No

Those around you were motivating you to undergo the operation.

○ Strongly Disagree      ○ Disagree       ○ Neutral          ○ Agree             ○ Strongly Agree

Public opinion can make the decision about whether to undergo surgery to treat obesity difficult.

○ Strongly Disagree      ○ Disagree       ○ Neutral          ○ Agree             ○ Strongly Agree

How does public opinion about obesity surgery affect individuals considering it?

○ Fear of negative reactions from others

○ Fear of discrimination and prejudice

○ Low self-esteem and self-confidence

○ Other: ____________

4  - Bariatric Surgery

Most of those who had the procedure as a treatment for obesity failed to follow a healthy diet and exercise.

○ Strongly Disagree      ○ Disagree       ○ Neutral          ○ Agree            ○ Strongly Agree

Most bariatric surgeries are limited only to lazy individuals.

○ Strongly Disagree      ○ Disagree       ○ Neutral          ○ Agree            ○ Strongly Agree

People who had bariatric surgery had other options available to them to lose weight.

○ Strongly Disagree      ○ Disagree       ○ Neutral          ○ Agree            ○ Strongly Agree

Surgery to treat obesity is the easiest way to lose weight instead of putting in the effort by following a diet and exercising.

○ Strongly Disagree      ○ Disagree       ○ Neutral          ○ Agree            ○ Strongly Agree

It is wrong for young men/women to undergo surgery to treat obesity.

○ Yes (in all situations)

○ Yes (In some cases)

○ No

It is important to address public opinion regarding bariatric surgery.

○ Strongly Disagree      ○ Disagree       ○ Neutral          ○ Agree            ○ Strongly Agree

Bariatric surgery is a cosmetic surgery.

○ Strongly Disagree      ○ Disagree       ○ Neutral          ○ Agree            ○ Strongly Agree

The quality of life of people who are obese and have undergone bariatric surgery is likely to be better than that of people who are obese and have not had the procedure.

○ Strongly Disagree      ○ Disagree       ○ Neutral          ○ Agree            ○ Strongly Agree

Those who underwent the surgery to treat obesity should be congratulated.

○ Strongly Disagree      ○ Disagree       ○ Neutral          ○ Agree            ○ Strongly Agree

Health insurance should cover the cost of the operation to facilitate access to it.

○ Strongly Disagree      ○ Disagree       ○ Neutral          ○ Agree            ○ Strongly Agree
